# EHPnet: Global Alliance for Improved Nutrition

**Published:** 2007-12

**Authors:** Erin E. Dooley

A third of the world’s population lacks sufficient vitamins and minerals in their diet. Each year nutritional deficiencies contribute to the deaths of 1 million children under the age of 5 years and 50,000 women during or just after childbirth. Such conditions also commonly cause mental and physical health problems such as learning disabilities, birth defects, and blindness. In addition, $6 billion dollars in lost adult work performance will add up annually due to malnutrition, significantly impacting the economies of developing countries. At a special 2002 UN session on child health, the Global Alliance for Improved Nutrition (GAIN) was formed to address the problems of inadequate diet in at-risk populations using food fortification, as described at **http://www.gainhealth.org/**.

To date GAIN has had three rounds of grant funding, with 18 projects in food fortification and infant/young child nutrition aiming to improve the nutrition of almost 600 million people. The Food Fortification subsection of the Our Programs page outlines the different levels of GAIN grants and initiatives in this area. Project leaders are working to establish and strengthen national food fortification programs in several developing countries with efforts to fortify such products as cooking oils, maize meal, wheat flour, and sauces. Smaller-scale targeted projects focus on four main program areas: schools, maternal and child health, populations affected with HIV/AIDS, and policy development. GAIN also supports global initiatives, one of which focuses on fortification of food that is being distributed to refugees, internally displaced persons, and other vulnerable groups.

GAIN explains the direction of its work in the Why Fortification? portion of its website, including a brief discussion of why GAIN has decided to channel its resources into fortification along with scientific data to support this move. Overviews of the five primary micronutrient deficiencies—vitamin A, iodine, folate, iron, and zinc—discuss how these nutrients are used in the body, how deficiencies in each impact health, and the primary sources of the nutrients in food. Four major strategies for eliminating nutritional deficiencies are laid out on the Possible Responses page along with the pros and cons of each.

